# Non-Coding RNAs in Cancer Liquid Biopsy: From Regulatory Networks to Functional Biomarkers for Precision Oncology

**DOI:** 10.3390/genes17080847

**Published:** 2026-07-23

**Authors:** Salvatore Pernagallo, Veronica Tisato, Donato Gemmati

**Affiliations:** 1 Department of Environmental and Prevention Sciences, University of Ferrara, 44121 Ferrara, Italy; 2 Department of Translational Medicine, University of Ferrara, 44121 Ferrara, Italy; veronica.tisato@unife.it (V.T.); d.gemmati@unife.it (D.G.)

**Keywords:** non-coding RNA, liquid biopsy, cancer, microRNA, long non-coding RNA, circular RNA, extracellular vesicles, precision oncology, PCR-free detection

## Abstract

Liquid biopsy is now an established component of precision oncology, and its clinical implementation to date has been led by cell-free DNA (cfDNA) and circulating tumor DNA (ctDNA) assays. These assays report genomic alterations, yet they may be limited by low tumor fraction, reduced shedding in early disease, and incomplete representation of dynamic tumor biology. Non-coding RNAs (ncRNAs), including microRNAs (miRNAs), long non-coding RNAs (lncRNAs), circular RNAs (circRNAs), and emerging small or poorly annotated RNA species, provide a complementary layer because they can reflect regulatory programs, tissue injury, immune modulation, metastatic communication, and therapeutic pressure. This review explores circulating and extracellular vesicle (EV)-associated ncRNAs as functional readouts in cancer liquid biopsy. We discuss their biological origin, carrier state, biofluid context, clinical applications, analytical technologies, artificial intelligence (AI)-assisted integration, standardization barriers, and regulatory requirements. The technology discussion considers sequencing, targeted amplification, and emerging direct or polymerase chain reaction (PCR)-free strategies as complementary translational routes for reliable ncRNA measurement. We propose that ncRNAs should not be viewed as alternatives to ctDNA, but as potential functional biomarkers that can link tumor genotype, regulatory state, and clinical phenotype within integrated multi-analyte precision oncology.

## 1. Introduction: Liquid Biopsy at a Turning Point

Liquid biopsy has entered routine clinical practice in precision oncology in selected settings [1,2,3,4]. By analyzing tumor- and host-derived material released into body fluids, it enables dynamic and repeatable molecular assessment without exclusive dependence on tissue sampling. Its clinical value is currently most evident for cell-free DNA (cfDNA) and circulating tumor DNA (ctDNA) assays, which are used to identify actionable genomic alterations, support companion diagnostic decisions, monitor molecular evolution, and guide treatment selection in advanced cancer [5,6,7,8,9,10,11,12]. Nevertheless, the maturity of liquid biopsy remains highly dependent on tumor type, clinical indication, analyte class, and analytical platform.

This clinical progress has been driven largely by DNA-based profiling, but a DNA-centered view captures only part of cancer biology. ctDNA is highly informative about the genomic architecture of a tumor, including sequence variants, copy-number changes, structural rearrangements, methylation patterns, and molecular residual disease signatures [13,14,15,16,17,18,19]. However, it does not fully describe the regulatory state of tumor cells, the behavior of the tumor microenvironment, the host inflammatory response, or the functional consequences of therapy-induced selective pressure. Moreover, ctDNA detection can remain challenging in early-stage disease, low-shedding tumors, minimal residual disease (MRD) settings, and anatomical contexts in which tumor-derived DNA is poorly represented in peripheral blood [20,21,22,23,24,25].

Non-coding RNAs (ncRNAs) are among the most informative of these complementary sources. Although they do not encode proteins, they regulate gene expression, chromatin organization, RNA stability, translation, cell signaling, and intercellular communication. In cancer, ncRNAs contribute to proliferation, apoptosis resistance, invasion, angiogenesis, immune evasion, metabolic rewiring, stemness, metastatic colonization, and drug resistance [26,27,28,29,30,31,32,33,34,35,36,37,38,39]. Their detection in plasma, serum, urine, saliva, stool, cerebrospinal fluid, and extracellular vesicles (EVs) therefore offers information that differs from ctDNA: evidence of tumor-associated molecular release, together with insight into biological programs active within the tumor-host system [40,41,42,43,44,45,46,47,48,49,50,51,52,53,54,55,56]. Indeed, the ncRNA literature in cancer diagnostics is already substantially larger and growing faster than that on cfDNA or ctDNA, although this research volume has not yet translated into a comparable number of regulatory-approved or guideline-endorsed clinical assays.

**Figure 1:**
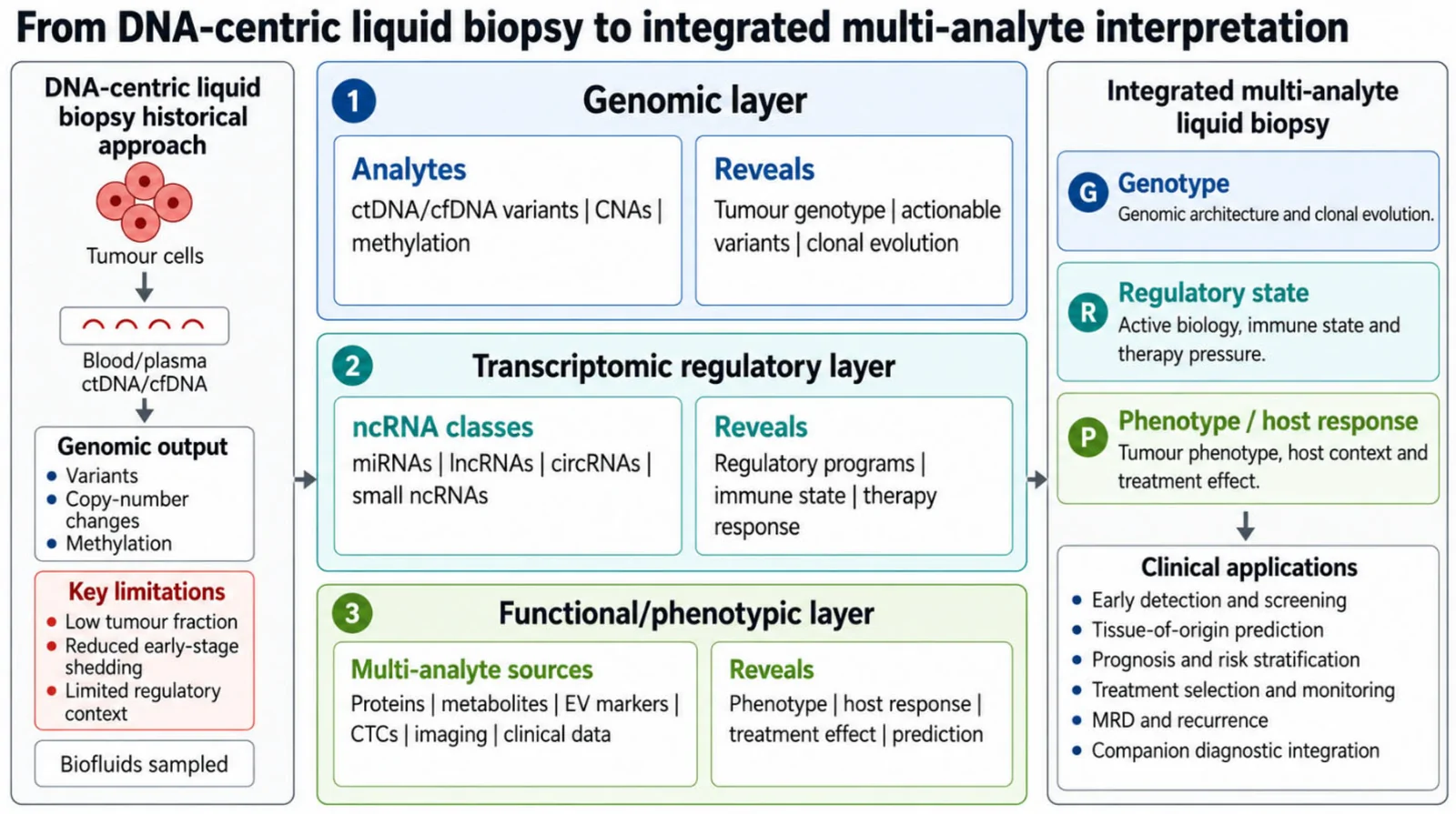
From DNA-centric to integrated multi-analyte liquid biopsy. The left panel summarizes the conventional DNA-centric approach, in which blood/plasma ctDNA/cfDNA analysis generates genomic outputs such as sequence variants, copy-number changes, and methylation patterns, while highlighting key limitations including low tumor fraction, reduced early-stage shedding, and limited regulatory context. The central panel illustrates an expanded layered framework integrating genomic information with an ncRNA-enriched transcriptomic regulatory layer and a functional/phenotypic layer. The right panel shows how genotype, regulatory state, and phenotype/host-response information can be combined to support precision oncology applications, including early detection, tissue-of-origin prediction, prognosis and risk stratification, treatment monitoring, minimal residual disease (MRD)/recurrence assessment, and companion diagnostic integration. Abbreviations: cfDNA, cell-free DNA; ctDNA, circulating tumor DNA; CTCs, circulating tumor cells; EV, extracellular vesicle; ncRNA, non-coding RNA.

The central argument of this review is that ncRNAs should be interpreted as functional readouts of cancer liquid biopsy [40,41,42,43,44,45]. They are not merely additional markers for expanded panels, but molecular reporters of tumor activity, host response, immune modulation, tissue damage, metastatic communication, and therapeutic pressure. The following sections therefore examine how ncRNAs can fill the functional gaps left by ctDNA by adding information on carrier state, biofluid context, biological interpretation, clinical intended use, analytical technology, computational integration, and regulatory translation. As liquid biopsy evolves from single-analyte testing toward multi-analyte and multi-omic diagnostic systems, clinically useful assays will increasingly integrate genomic, transcriptomic, proteomic, vesicular, cellular, imaging, and computational information [20,21,22,23,24,25]. This integrated framework is summarized in Figure 1, in which ncRNAs function as the transcriptomic regulatory layer linking tumor genotype, regulatory state, and clinical phenotype.

### Scope and Approach

This article is a narrative review. Relevant literature was identified through targeted searches of PubMed and Google Scholar up to June 2026, using combinations of terms including ‘non-coding RNA’, ‘microRNA’, ‘long non-coding RNA’, ‘circular RNA’, ‘extracellular vesicles’, ‘liquid biopsy’, ‘cancer’, ‘biomarker’, and ‘precision oncology’. This was supplemented by manually searching reference lists and recent reviews in the field. Studies were prioritised for inclusion based on their relevance to ncRNA biology, carrier state, clinical application, analytical technology and regulatory translation in cancer liquid biopsy rather than through a predefined systematic protocol or formal quality-scoring framework. The aim of this narrative approach is to synthesise a conceptual framework linking ncRNA biology to clinical. Readers seeking systematic evidence synthesis on specific ncRNA-biomarker associations should consult the dedicated systematic reviews and meta-analyses cited throughout this article.

## 2. From Liquid Biopsy to the Liquid Transcriptome

Liquid biopsy is often discussed as a single analytical concept, but the molecular entities measured in body fluids differ markedly in origin, stability, abundance, and clinical meaning. ctDNA is released predominantly through cell death [13,14,15,16,17,18,19], whereas circulating RNA can arise from both passive release and active secretion [40,41,42,43,44,45,46,47,48,49,50,51,52]. ncRNAs may circulate as free or protein-bound molecules, in association with lipoproteins, within EVs, in platelets, or as part of apoptotic and necrotic material. This diversity complicates assay design but also expands the biological information available from liquid biopsy.

Circulating ncRNAs can therefore be viewed as part of a liquid transcriptome: a composite molecular signal generated by tumor cells, stromal cells, immune cells, endothelial cells, blood cells, and injured tissues [51,52]. This signal is not equivalent to the transcriptome of the primary tumor. Instead, it is a dynamic mixture shaped by tumor burden, anatomical location, vascularization, inflammation, therapy, sample handling, and the carrier in which each RNA species is found [57,58].

Carrier state is central to interpretation. The same ncRNA may have different diagnostic and biological meaning when measured in total plasma, EVs, platelets, protein complexes, lipoprotein fractions, or tissue-proximal fluids [43,44,45,59,60,61,62,63,64,65,66,67]. For example, a miRNA released during tissue injury may indicate cell damage, whereas the same miRNA enriched in tumor-derived vesicles may reflect active intercellular communication. Similarly, platelet-associated RNA signatures may report tumor-induced platelet education, whereas EV-associated RNA may reflect selective packaging and secretion by tumor or stromal cells [40,41,42,43,44,45,46,47,48,49,50,51,52,59,60,61,62,63,64,65,66,67].

This distinction has practical consequences for clinical translation [6]. Assays measuring total circulating RNA may offer simplicity and sensitivity, but they may integrate signals from multiple tissues and physiological processes. Carrier-enriched assays may improve biological specificity, but they introduce additional pre-analytical and technical variables [56,68,69,70,71,72,73,74,75]. The optimal strategy should therefore be determined by the intended clinical use: early detection may benefit from broad, high-sensitivity signatures, whereas treatment monitoring and resistance prediction may require carrier-defined signals linked more directly to tumor biology [76,77,78,79].

## 3. Non-Coding RNA Classes in Cancer Liquid Biopsy: From Molecular Categories to Clinical Functions

ncRNAs include a heterogeneous collection of molecules that differ in size, biogenesis, structure, stability, and function [26,27,28,29,30,31,32,33,34,35,36,37,38,39]. In cancer liquid biopsy, the most frequently studied classes include miRNAs, long non-coding RNAs (lncRNAs), circular RNAs (circRNAs), PIWI-interacting RNAs (piRNAs), tRNA-derived fragments (tRFs), small nucleolar RNAs (snoRNAs), small nuclear RNAs (snRNAs), Y RNAs, and other poorly annotated or orphan ncRNAs [53,54,55,56,57,80,81,82,83,84,85,86,87,88,89,90,91,92,93,94,95,96,97,98,99,100,101]. For clinical translation, it is most useful to organize these classes according to the type of biological and clinical information they provide (Table 1).

### 3.1. MicroRNAs: Stable and Actionable Small-RNA Biomarkers

miRNAs are the most established ncRNA biomarkers in liquid biopsy [40,41,42,43,44,45,68,69,70,71,72]. Their small size, relative stability, disease-associated expression patterns, and compatibility with targeted assays have made them highly attractive for cancer detection, prognosis, and monitoring. In many tumors, miRNA signatures reflect cellular pathways linked to proliferation, apoptosis, epithelial–mesenchymal transition (EMT), angiogenesis, immune regulation, and treatment resistance [26,27,28,29,30,31,32,93]. Their major translational challenge is not biological relevance, but reproducibility across cohorts, specimen types, extraction methods, normalization strategies, and analytical platforms [53,54,55,56,73,74,75]. Beyond oncology, miRNAs have also been described as epigenetic regulators of tissue remodeling in other conditions, such as post-infarction cardiac wall remodeling, further supporting their broader mechanistic relevance as functional biomarkers [102].

### 3.2. Long Non-Coding RNAs: Tissue Specificity and Regulatory Depth

lncRNAs offer a different type of promise. Many lncRNAs show tissue- or cancer-type-enriched expression and can regulate transcription, chromatin state, splicing, RNA stability, and signaling networks [36,37,38,39,80,81,82,83]. Their cancer specificity may support tumor classification and prognostic stratification, but their lower abundance and greater susceptibility to degradation can complicate circulating detection. Extracellular-vesicle enrichment, targeted amplification, or direct capture strategies are especially relevant for their reliable clinical measurement [103,104,105,106,107,108,109,110,111,112,113].

### 3.3. Circular RNAs: Structural Stability and Biofluid Robustness

circRNAs are attractive liquid biopsy candidates because their covalently closed structure confers resistance to exonuclease degradation [84,85,86,87,88,89,90]. This structural stability is highly advantageous in harsh biofluid environments such as plasma, serum, urine, saliva, and stool. Functionally, circRNAs can act as miRNA sponges, interact with RNA-binding proteins, modulate transcription, and in some cases encode functional peptides [84,85,86,87,88,89,90,114,115,116,117,118,119,120]. Their presence in EVs and tissue-specific expression patterns make them promising for robust biomarker development, although standardized annotation and quantification remain key operational challenges. Similarly, circRNAs have been implicated as epigenetic regulators of blood–brain barrier integrity and have shown disease-specific expression profiles in non-oncological conditions such as multiple sclerosis, illustrating the broader regulatory versatility of this RNA class across disease contexts [121,122].

### 3.4. Emerging Small and Poorly Annotated ncRNAs: High-Dimensional Cancer Fingerprints

Emerging small RNA classes, including tRFs, piRNAs, snoRNAs, Y RNA fragments, and poorly annotated ncRNAs, are expanding the liquid biopsy landscape [57,91,94,95,96,97,98,99,100,101]. These molecules provide high-dimensional cancer fingerprints that are not captured by conventional miRNA panels. Some are particularly useful in artificial intelligence (AI)-driven signatures where diagnostic information emerges from complex global patterns rather than from single canonical biomarkers [74,75,76,77,78,79]. However, this strength creates a translational risk: signatures may be statistically powerful but biologically opaque unless supported by rigorous validation and mechanistic interpretation.

Taken together, these ncRNA classes should not be treated as competing biomarker families, but as complementary molecular layers. miRNAs provide stability and assay practicality; lncRNAs provide regulatory and tissue-specific information; circRNAs offer structural robustness; EV-associated RNAs provide carrier-defined specificity; and emerging small RNAs offer high-dimensional discriminatory power. The most clinically useful liquid biopsy assays will likely combine selected RNA classes according to the intended use rather than relying on a single category.

## 4. Carrier-Aware ncRNA Biomarkers: Why Molecular Context Matters

The clinical value of a circulating ncRNA depends on its sequence and abundance, as well as its biological context. Body fluids contain a complex mixture of RNA carriers, including ribonucleoprotein complexes, lipoproteins, EVs, platelets, apoptotic bodies, cellular debris, and intact rare cells [40,41,42,43,44,45,46,47,48,49,50,51,52,59,60,61,62,63,64,65,66,67]. Each carrier may reflect a different biological process and may require a different analytical workflow. For this reason, future liquid biopsy studies should explicitly define whether they aim to measure total circulating ncRNA, a carrier-enriched fraction, or a tissue-proximal RNA signal [123,124,125,126,127].

Cell-free and protein-bound ncRNAs offer analytical accessibility because they can be measured directly from plasma or serum without complex vesicle enrichment. However, they may also reflect systemic tissue injury, blood cell contamination, inflammation, or sample handling effects [43,44,56,68,69,70,71,72]. Lipoprotein-associated RNAs may provide additional biological information because lipoproteins participate in intercellular transport, metabolism, and inflammatory signaling. Platelet-associated RNAs form another relevant compartment: tumor-educated platelets can acquire altered RNA profiles through interaction with cancer cells and the tumor microenvironment, potentially providing indirect but informative signatures of malignancy [45,66,67].

EV-associated ncRNAs occupy an especially important position in the field. EVs can protect RNA cargo from degradation and may reflect selective packaging by tumor cells, stromal cells, immune cells, and endothelial cells [46,47,48,49,50,59,60,61,62,63,64,65]. Vesicle-associated RNAs may therefore function both as biomarkers and as mediators of tumor-microenvironment communication. However, the same biological richness creates technical challenges. EV isolation methods differ in yield, purity, scalability, and compatibility with clinical workflows [123,124,125,126,127]. Without rigorous reporting and characterization, apparent EV-ncRNA biomarkers may be confounded by co-isolated protein complexes, lipoproteins, or non-vesicular RNA carriers.

**Figure 2:**
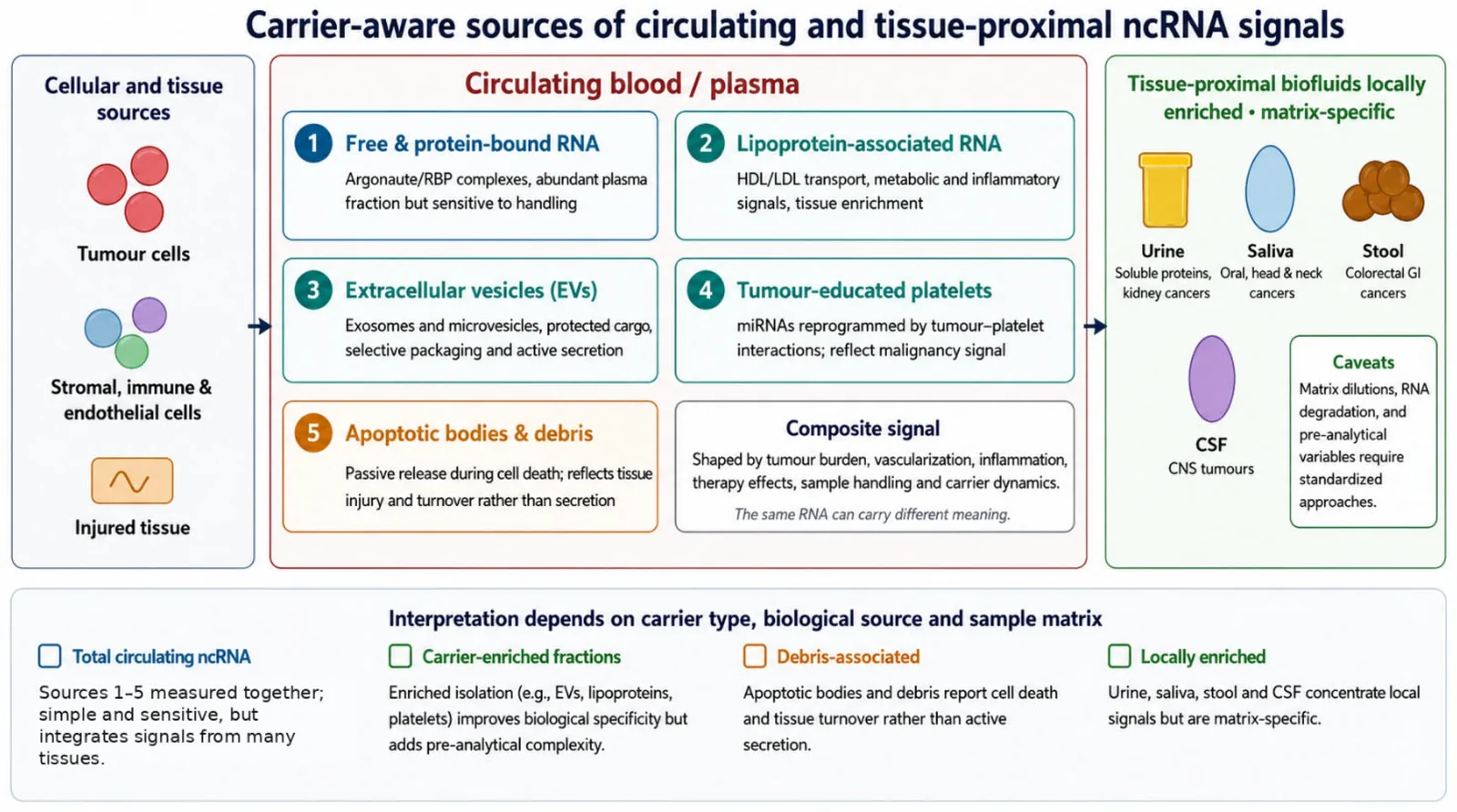
Carrier-aware sources of circulating and tissue-proximal ncRNA signals. The schematic links cellular and tissue sources to major RNA-containing compartments detectable in blood/plasma, including free or protein-bound RNA, lipoprotein-associated RNA, extracellular vesicles (EVs), tumor-educated platelets, apoptotic bodies, and cellular debris. It also summarizes locally enriched biofluids, including urine, saliva, stool, and cerebrospinal fluid (CSF), and highlights that the same ncRNA may have different meaning depending on carrier type, biological source, sample matrix, and pre-analytical handling. Abbreviations: CSF, cerebrospinal fluid; EV, extracellular vesicle; ncRNA, non-coding RNA.

Tissue-proximal biofluids add another dimension. Urine may be especially relevant for urological cancers, saliva for head and neck malignancies, stool for colorectal cancer and gastrointestinal bleeding-related signals, and cerebrospinal fluid for central nervous system tumors [49,50,128,129,130,131,132]. These biofluids can enrich local disease signals that may be diluted in peripheral blood, but they also introduce matrix-specific inhibitors, degradation processes, and pre-analytical variables [58,73]. Figure 2 summarizes this carrier-aware and matrix-aware logic for the rational design of ncRNA liquid biopsy assays.

## 5. Biological Interpretation: ncRNAs as Functional Disease Sensors

A major conceptual advantage of ncRNAs is their ability to report biological activity. Whereas ctDNA primarily reflects genomic alterations and tumor fraction [13,14,15,16,17,18,19,20,21,22,23,24,25], ncRNAs can provide information about regulatory programs and phenotypic states [26,27,28,29,30,31,32,33,34,35,36,37,38,39,40,41,42,43,44,45]. In this sense, circulating ncRNAs may act as functional disease sensors. They can report tumor presence, tissue-of-origin signals, malignant aggressiveness, host response, immune modulation, metastatic competence, and therapeutic pressure.

For early detection, ncRNA signatures may be useful when tumor DNA shedding is low or intermittent [40,41,42,43,44,45]. For prognosis, specific ncRNA patterns may reflect aggressive biological programs, including EMT, angiogenesis, invasion, stemness, and metastatic niche preparation [26,27,28,29,30,31,32,33,34,35,36,37,38,39,68,69,70,71,72]. For treatment monitoring, changes in circulating ncRNAs may capture therapy-induced stress, immune activation, tumor cell death, or emerging resistance. In immuno-oncology, EV-associated ncRNAs are of particular interest because they may participate in immune escape, antigen presentation modulation, cytokine signaling, and the regulation of immune checkpoint pathways [59,60,61,62,63,133].

The same biological sensitivity that makes ncRNAs attractive also requires careful interpretation. Many ncRNAs are not cancer-exclusive and may be influenced by age, sex, inflammation, infection, medication, tissue injury, metabolic disease, and lifestyle [56,68,69,70,71,72]. Therefore, clinically useful ncRNA signatures should not be interpreted simply as tumor markers, but as disease-context markers. Their value will be highest when the biological question is clearly defined and when comparator populations reflect realistic clinical use rather than idealized health controls [6,73,74,75,76,77,78,79].

A further implication is that ncRNA biomarkers may be most informative when interpreted as dynamic, context-dependent signals rather than as fixed binary indicators of tumor presence. In longitudinal sampling, a change in an ncRNA signature may reflect a shift in tumor biology, treatment-induced tissue injury, immune activation, or remodeling of the tumor microenvironment before these processes become radiologically apparent [40,41,42,43,44,45,46,47,48,49,50,51,52,59,60,61,62,63]. Conversely, stable baseline differences between patients may capture host biology, organ-specific vulnerability, or chronic inflammatory states that influence cancer risk and treatment tolerance. This dual tumor-host origin should be considered a strength rather than a limitation, provided that the intended clinical question is explicit.

Because many ncRNAs, and miRNAs in particular, act by post-transcriptionally repressing specific messenger RNA (mRNA) targets, an additional layer of validation can be obtained by confirming that a candidate circulating ncRNA produces the expected downstream effect on its predicted or experimentally established mRNA targets. Approaches such as luciferase 3′-untranslated-region reporter assays, RNA-induced silencing complex pulldown, and paired ncRNA-mRNA expression analysis in matched tissue or cellular models can demonstrate that a circulating biomarker candidate is statistically associated with cancer status and mechanistically active within a relevant regulatory pathway [134]. This distinction matters clinically: an ncRNA that is merely correlated with disease may still be useful as an empirical biomarker, but an ncRNA with confirmed downstream mRNA regulation offers a stronger rationale for biological plausibility and can support the interpretation of longitudinal changes during treatment. We therefore encourage, where feasible, that candidate ncRNA biomarkers be accompanied by orthogonal functional validation of their predicted mRNA targets, in addition to standard analytical and clinical validation.

For example, an ncRNA pattern used for early detection should be optimized for cancer-associated discrimination, whereas an ncRNA pattern used for treatment monitoring may legitimately incorporate tumor injury, immune response, and organ toxicity signals. The functional interpretation of ncRNAs therefore depends on integrating molecular abundance with carrier state, sampling time point, clinical context, and comparator population [6,68,69,70,71,72,73,74,75,76,77,78,79,123,124,125,126,127,128,129,130,131,132].

## 6. Clinical Applications in Precision Oncology

The clinical development of ncRNA liquid biopsy should be organized around intended use. A biomarker signature designed for population screening has different performance requirements from a signature designed for recurrence monitoring, therapy response, or resistance prediction [6,74,75]. Early cancer detection requires high sensitivity for low-burden disease and high specificity in clinically relevant populations [20,21,22,23,24,25]. Tumor classification and tissue-of-origin prediction require signatures that distinguish cancer types and account for inflammatory or benign disease controls. Prognostic signatures require association with clinically meaningful outcomes such as progression-free survival, overall survival, metastatic relapse, or treatment failure [58,66,67,128,129,130,131,132].

In therapy selection, ncRNAs may be used to infer pathway activation, immune state, or resistance biology. EV-associated ncRNAs could complement ctDNA by reporting tumor-microenvironment communication or immune escape [59,60,61,62,63,64,65,66,67]. In MRD and recurrence monitoring, ncRNAs may provide complementary information about when ctDNA is undetectable or when active tumor biology precedes radiological progression [13,14,15,16,17,18,19,20,21,22,23,24,25]. Finally, biofluid-specific applications may allow disease-proximal measurements: urine for bladder, prostate, and kidney cancers; saliva for oral and head and neck cancers; stool for colorectal cancer; and plasma or serum for systemic profiling [128,129,130,131,132]. Concrete examples illustrate this potential: profiling of tumor-educated platelet RNA distinguished patients with cancer from healthy individuals with 96% accuracy and identified the tissue of origin across six tumor types with 71% accuracy [66], while a particle-swarm-optimized platelet RNA classifier detected non-small-cell lung cancer with areas under the curve of 0.94 and 0.89 in late- and early-stage validation cohorts, respectively [67]. Such performance is encouraging but still requires confirmation in prospective, intended-use populations with appropriate benign and inflammatory comparators.

Table 2 summarizes the main intended-use scenarios and the corresponding validation focus. These clinical applications should not be collapsed into a single generic biomarker claim. A screening assay must perform in asymptomatic or enriched-risk populations in which disease prevalence is low and false positives may generate unnecessary diagnostic procedures [20,21,22,23,24,25]. A diagnostic classifier must distinguish cancer from benign, inflammatory, or pre-malignant conditions that are likely to appear in the same clinical pathway. A prognostic assay should add information beyond established clinicopathological variables, whereas a monitoring assay should demonstrate that longitudinal molecular changes anticipate or improve clinical decision-making compared with imaging, conventional biomarkers, or ctDNA alone. For therapy response and resistance, the most informative ncRNA signals may be those linked to pathway activity, immune state, or tumor-host communication rather than tumor burden alone [58,59,60,61,62,63,64,65,66,67]. This intended-use discipline is essential for designing appropriate comparator cohorts, statistical endpoints, sampling intervals, and clinical decision thresholds [6,73,74,75,76,77,78,79].

It is important to emphasize that, at present, most ncRNA-based signatures described in this review remain at the discovery or early clinical-validation stage. Robust confirmation in adequately powered, prospective clinical trials with predefined intended-use populations, comparator groups, and clinical endpoints is still needed before these applications can be considered clinically validated diagnostic or prognostic tools rather than promising investigational candidates [6].

## 7. Technologies for ncRNA Liquid Biopsy Translation

The transition from biomarker discovery to clinical implementation depends strongly on analytical technology. RNA sequencing and small-RNA sequencing remain powerful discovery tools because they provide broad profiling capacity and can identify canonical and non-canonical RNA species [76,77,78,79]. Targeted sequencing can improve depth and reduce cost, but it still requires careful library preparation, bioinformatic normalization, and batch-effect control. Reverse-transcription quantitative polymerase chain reaction (RT-qPCR) remains widely used because it is sensitive, accessible, and suitable for targeted validation, but it can be affected by reverse-transcription efficiency, primer specificity, amplification bias, and normalization uncertainty [73,75]. Digital polymerase chain reaction (dPCR) can improve absolute quantification and sensitivity for selected targets, although its multiplexing capacity remains more limited than sequencing or bead-based platforms [74]. These established technologies remain essential, but they do not eliminate the need for more direct workflows that reduce sample loss, target conversion bias, and amplification-dependent artifacts.

Direct and polymerase chain reaction (PCR)-free strategies are relevant to ncRNA liquid biopsy because they may reduce dependence on RNA extraction, reverse transcription, and amplification, thereby limiting target loss and analytical bias. One example of this broader technology class is Dynamic Chemical Labelling (DCL), which uses target-guided chemistry on modified peptide nucleic acid probes to convert sequence recognition into optical, chemiluminescent, digital, or bead-based signals. Published proof-of-concept studies have reported direct detection of selected miRNAs in non-oncological contexts and compatibility with multiplex bead-based readouts [135,136,137,138,139,140,141,142,143,144,145,146], but DCL has not yet been validated in oncological cohorts; a recent dedicated review discusses the broader landscape of amplification-free microRNA testing specifically in cancer [147]. As with the other emerging direct-read approaches discussed below, its translational value in cancer liquid biopsy will depend on independent, investigator-led validation in clinically relevant biofluids, using locked signatures and head-to-head comparison with established sequencing, PCR-based, and hybridization-based platforms.

Other emerging direct or low-amplification approaches should be considered alongside chemical-ligation strategies. Clustered regularly interspaced short palindromic repeats (CRISPR)/CRISPR-associated (Cas)-based diagnostics, including Specific High-Sensitivity Enzymatic Reporter UnLOCKing (SHERLOCK) and DNA Endonuclease-Targeted CRISPR Trans Reporter (DETECTR), exploit programmable nucleic-acid recognition and collateral reporter cleavage to generate sensitive sequence-specific readouts [148,149,150]. These systems offer attractive features for liquid biopsy, including programmability, portability, and potential point-of-care formats. At the same time, many implementations still rely on pre-amplification, and their quantitative performance in complex cancer biofluids, carrier-enriched fractions, stool, urine, or low-abundance ncRNA settings requires further analytical and clinical validation.

Surface-enhanced Raman spectroscopy (SERS) and plasmonic biosensors provide another complementary route for sensitive and potentially multiplexed nucleic-acid detection. Recent SERS-based liquid biopsy reviews and miRNA-focused biosensors highlight the capacity of these platforms to analyze circulating nucleic acids, extracellular vesicles, proteins, and miRNA targets with low sample consumption and optical multiplexing [151,152]. Their translational challenges include substrate reproducibility, probe design, matrix interference, spectral standardization, calibration across instruments, and the need for transparent machine-learning pipelines when spectral classifiers are used. Nanopore and direct RNA sequencing approaches may also contribute to future RNA liquid biopsy by enabling longer-read, isoform-level, and potentially epitranscriptomic information; however, input requirements, short-RNA capture, error profiles, and robustness in clinical biofluids remain important limitations [153].

Overall, no single technology is optimal for all stages of ncRNA liquid biopsy development. Discovery may require sequencing or broad profiling, whereas clinical implementation may favor targeted, standardized, cost-effective, and reproducible platforms. The most appropriate assay should be selected according to intended use, target number, required sensitivity, sample matrix, throughput, regulatory pathway, laboratory infrastructure, and the degree to which the platform can be externally validated in real-world clinical cohorts. Table 3 summarizes the comparative advantages and limitations of the technologies discussed above.

## 8. Artificial Intelligence and Multi-Analyte Integration

High-dimensional ncRNA data are well suited to computational modeling because diagnostic information may arise from patterns distributed across many RNA species rather than from single biomarkers [74,75,76,77,78,79,154]. In current liquid biopsy and precision-oncology studies, model classes commonly include regularized regression, random forests, support vector machines, gradient boosting, and deep-learning architectures, with graph-based or multi-view models becoming increasingly relevant for multi-omic integration. AI and machine learning can integrate canonical ncRNAs, poorly annotated RNAs, ctDNA, proteins, EV markers, imaging, and clinical metadata into composite classifiers [20,21,22,23,24,25,155,156]. This approach may be especially useful for early detection, tissue-of-origin prediction, and complex response phenotypes such as immunotherapy benefit or resistance [157,158]. As a proof of concept, an AI-normalized cell-free RNA (liquid transcriptome) classifier separated patients with solid tumors from controls with an area under the curve of 0.82, and distinguished myeloid and lymphoid neoplasms with areas under the curve of 0.86 and 0.79, respectively [156], illustrating both the promise and the still-preliminary nature of transcriptome-wide AI classifiers prior to prospective validation.

The choice of computational approach should reflect both the structure of the input data and the clinical question being addressed. Classical regularized regression and tree-based ensemble methods, such as random forests and gradient boosting, remain widely used for moderate-sized, tabular ncRNA panels because they are relatively robust to small sample sizes, provide interpretable feature-importance rankings, and are less prone to overfitting than deep architectures when the number of features approaches or exceeds the number of samples [154,155]. Support vector machines and related margin-based classifiers have also been extensively applied to circulating RNA and platelet-derived signatures, including particle-swarm-optimized variants used for tumor-educated platelet classification [66,67]. Deep-learning architectures, including autoencoders, variational autoencoders, and transformer-based foundation models pretrained on large cell-free RNA compendia, are increasingly applied when the input data are high-dimensional, sparse, or benefit from learned latent representations that denoise technical noise and augment limited case numbers; recent examples include a generative model trained on poorly annotated circulating RNAs for early-stage lung cancer detection and a multimodal transformer-based foundation model integrating RNA sequence and cell-free abundance features across thousands of samples [159,160]. Graph-based and multi-view architectures are emerging for multi-omic integration, in which ncRNA, ctDNA, protein, and imaging features are combined through shared or aligned latent spaces rather than simple feature concatenation [155,156]. Regardless of model class, training data should be structured with clearly defined case–control or longitudinal designs, documented pre-processing and normalization pipelines, and pre-specified train/validation/test partitions that avoid information leakage across related samples; these methodological details are frequently under-reported in the ncRNA machine-learning literature and should be considered a minimum standard for future studies [154,158].

For clinical translation, the role of AI should be defined before model development rather than after statistical optimization [6,154]. In screening or diagnostic triage, algorithms may be expected to maximize sensitivity while maintaining acceptable specificity in heterogeneous populations; in monitoring or therapy-response settings, they may instead prioritize longitudinal change, calibration, and robustness to repeated measurements [74,75,76,77,78,79,155,156,157,158]. Models based on poorly annotated ncRNAs or multi-analyte inputs should therefore report area-under-the-curve metrics alongside calibration, decision thresholds, feature stability, missing-data handling, and performance across clinically relevant subgroups. Interpretability is also important: even when individual ncRNAs are not mechanistically characterized, the final classifier should remain biologically plausible, technically reproducible, and compatible with a predefined clinical action. In this sense, AI should be viewed as a translation tool for integrating complex ncRNA signals, not as a substitute for analytical validation, external replication, or intended-use evidence.

However, computational power does not replace biological and clinical rigor. Models trained on retrospective or highly selected cohorts may not generalize to real-world screening, diagnostic, or monitoring populations [6,154,158]. Overfitting, batch effects, population bias, and inadequate external validation remain major risks [74,75,76,77,78,79]. Future AI-based ncRNA liquid biopsy studies should therefore include locked signatures, independent validation cohorts, transparent reporting, clinically relevant controls, and predefined performance endpoints.

## 9. Pre-Analytical, Analytical, and Clinical Standardization

Standardization is one of the main barriers to ncRNA liquid biopsy translation. Pre-analytical variables include blood collection tube type, time to processing, centrifugation protocol, hemolysis, platelet contamination, freeze–thaw cycles, storage conditions, and biofluid-specific matrix effects [56,68,69,70,71,72,123,124,125,126,127]. Analytical variables include RNA extraction efficiency, carrier enrichment strategy, reverse transcription efficiency, amplification bias, sequencing depth, adapter ligation bias, normalization method, and inter-platform comparability [73,74,75,76,77,78,79]. Clinical variables include cohort size, case–control definition, benign disease controls, medication, inflammation, comorbidities, and longitudinal sampling design [6].

A related consideration is that ncRNA abundance is intrinsically more dynamic than ctDNA-based genomic signals. Whereas ctDNA reports comparatively static sequence-level alterations that are either present or absent, circulating ncRNA levels fluctuate continuously with tumor burden, inflammation, circadian rhythm, exercise, diet, and numerous non-malignant physiological states [56,68,69,70,71,72]. This wider and more labile dynamic range increases the risk of both false-positive signals, driven by non-cancer physiological variation, and false-negative signals, driven by assay-specific normalization artifacts that can mask true biological differences [161,162]. Rigorous ncRNA quantification therefore requires validated endogenous or spike-in normalization strategies, platform-specific dynamic-range characterization, and pre-specified statistical thresholds established in independent cohorts, rather than normalization procedures optimized post hoc on the discovery dataset itself [161,162]. Without these safeguards, apparent ncRNA biomarker differences may reflect normalization bias rather than genuine tumor biology, a risk that is more pronounced for ncRNAs than for the comparatively binary readouts of ctDNA-based assays.

For EV studies, reporting standards are particularly important because different EV enrichment methods can isolate different mixtures of vesicles, lipoproteins, protein complexes, and ribonucleoprotein particles [123,124,125,126,127]. Transparent reporting of sample handling, EV separation, particle characterization, RNA extraction, and normalization is essential for reproducibility. More broadly, ncRNA liquid biopsy studies should move from exploratory biomarker lists to locked signatures and prospectively defined clinical questions. Table 4 summarizes the key pre-analytical and analytical variables that should be controlled for reproducible ncRNA liquid biopsy development [6,73,74,75,76,77,78,79].

## 10. Regulatory and Intended Use Roadmap

The clinical translation of ncRNA liquid biopsy requires more than biomarker discovery [6]. A regulatory-grade development pathway should begin with a clearly defined intended use and clinical context. The intended use determines the target population, comparator, acceptable false-positive and false-negative rates, sample type, assay format, and validation endpoint. A screening assay, a diagnostic classifier, a prognostic test, and a therapy-monitoring tool require different evidence packages [74,75].

An important intended-use distinction that shapes both clinical value and regulatory burden is whether an ncRNA assay is developed as a triage test or as a stand-alone diagnostic or prognostic assay. A triage assay is intended to enrich a population for further investigation, for example selecting patients for confirmatory imaging, endoscopy, or tissue biopsy, and can tolerate a lower positive predictive value if it substantially reduces unnecessary downstream procedures and cost; its regulatory evidence package typically emphasizes sensitivity, negative predictive value, and impact on the downstream diagnostic pathway rather than stand-alone diagnostic accuracy [163,164]. A stand-alone assay intended to directly inform a clinical decision, such as treatment selection or a change in surveillance intensity, requires substantially more rigorous analytical and clinical validation, including demonstration of clinical utility, because clinicians and patients act on its result without an intermediate confirmatory step [163,164,165]. Most ncRNA signatures discussed in this review currently have evidence packages more consistent with a triage or hypothesis-generating role than with stand-alone diagnostic or prognostic use; explicitly defining this intended positioning early in assay development is essential for selecting an appropriate regulatory pathway, comparator population, and validation burden [163,164].

**Figure 3:**
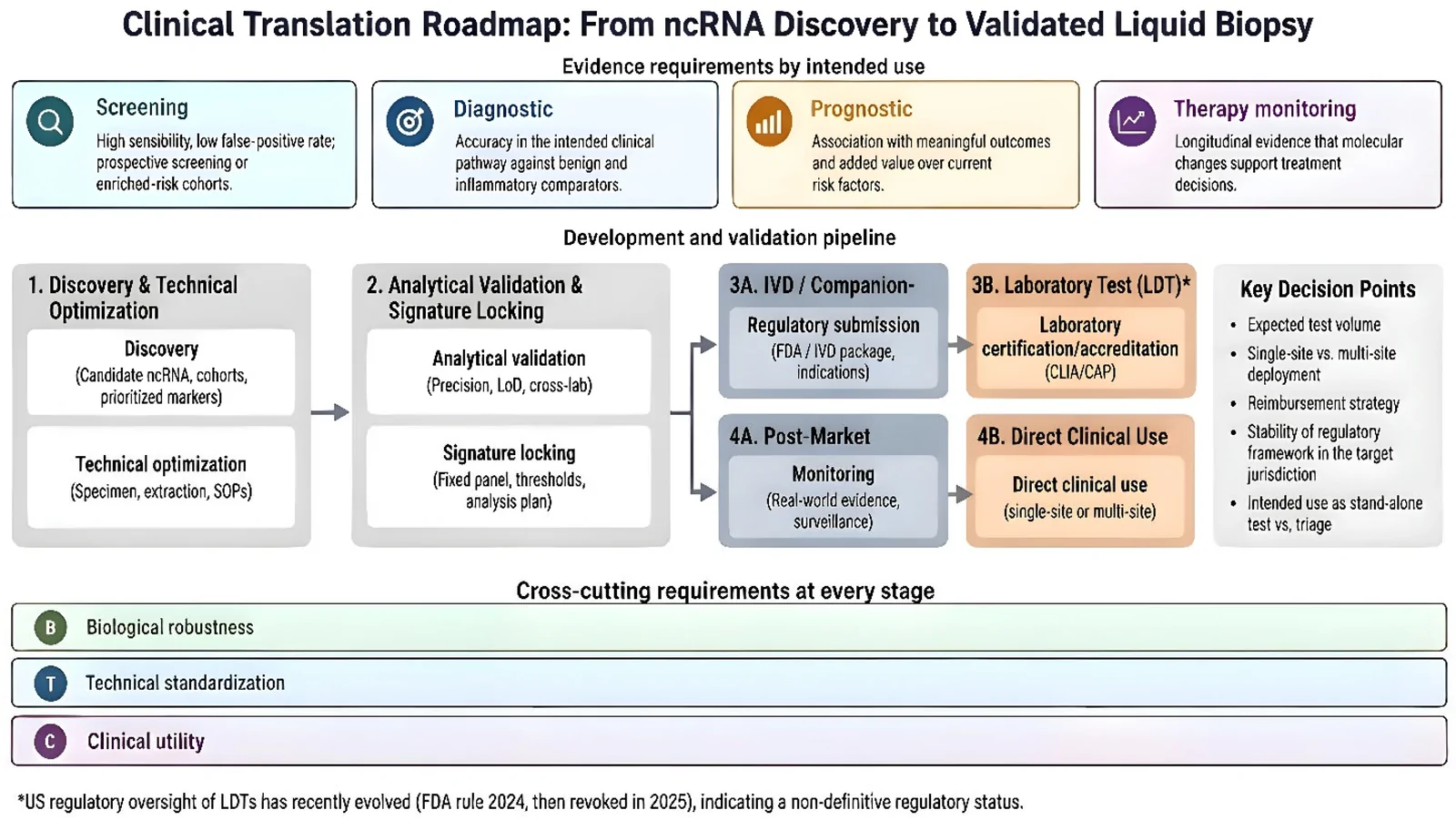
Clinical translation roadmap from ncRNA discovery to validated liquid biopsy. The figure links four intended-use contexts—screening, diagnostic classification, prognostic stratification, and therapy monitoring—to the main development steps required for clinical implementation. The central pipeline summarizes discovery, technical optimization, analytical validation, signature locking, and subsequent implementation through either a formal in vitro diagnostic (IVD)/companion-diagnostic route or a laboratory-developed test (LDT) route. The side panel highlights key decision factors, including expected test volume, single- versus multi-site deployment, reimbursement strategy, regulatory stability, and stand-alone versus triage use. The lower bars summarize cross-cutting requirements: biological robustness, technical standardization, and clinical utility. The note indicates that U.S. LDT oversight has recently changed, including the 2025 reversal of the 2024 FDA rule. This is a reminder that the regulatory pathway is still evolving. Abbreviations: CAP, College of American Pathologists; CLIA, Clinical Laboratory Improvement Amendments; FDA, U.S. Food and Drug Administration; IVD, in vitro diagnostic; LDT, laboratory-developed test; LoD, limit of detection; ncRNA, non-coding RNA.

A practical roadmap includes discovery, technical optimization, analytical validation, signature locking, retrospective clinical validation, prospective intended-use validation, health-economic assessment, regulatory submission, and post-market monitoring. Analytical validation should address sensitivity, specificity, precision, reproducibility, linearity, limit of detection, interference, stability, and matrix effects [73,74,75,123,124,125,126,127]. Clinical validation should demonstrate that the assay answers a clinically relevant question better than, or complementary to, existing standards of care [6,74,75]. Without this roadmap, ncRNA biomarkers risk remaining in the discovery literature without entering clinical practice. This intended-use-driven roadmap is illustrated in Figure 3. Because ncRNA assays may reach the clinic through different regulatory routes, it is useful to distinguish the laboratory-developed test (LDT) pathway from a formal in vitro diagnostic (IVD) submission pathway, such as premarket review by the U.S. Food and Drug Administration (FDA) or conformity assessment under the European In Vitro Diagnostic Regulation (IVDR). LDTs are designed, validated, and performed within a single certified clinical laboratory and can offer a faster route to clinical use, particularly for early-adoption or low-volume assays such as many current ncRNA signatures; however, oversight of LDTs has recently been in regulatory flux, illustrated by the FDA’s 2024 rule extending device-level oversight to LDTs, its vacatur by a federal court in 2025, and the FDA’s formal rescission of that rule later in 2025, which restored the pre-2024 policy of enforcement discretion over most LDTs [165]. Laboratories developing ncRNA assays as LDTs should nonetheless voluntarily adhere to analytical and clinical validation standards comparable to those expected for a formal IVD submission, given that the regulatory landscape may shift again. A formal IVD or companion-diagnostic submission requires a locked assay design, comprehensive analytical and clinical validation across each intended indication, and manufacture under a quality management system, but offers broader distribution, reimbursement pathways, and regulatory durability once approved [163,164]. Key decision points for a laboratory choosing between these pathways include projected test volume, single-site versus multi-site deployment, reimbursement strategy, the regulatory stability of the target jurisdiction, and whether the assay is intended as a stand-alone clinical decision tool or as a triage test with a lower evidentiary threshold. This LDT-versus-IVD distinction complements the intended-use-driven roadmap illustrated in Figure 3 and should be considered explicitly when planning the regulatory strategy for a new ncRNA liquid biopsy assay.

## 11. Future Perspective: Toward Functional, Multi-Analyte Liquid Biopsy

The future of cancer liquid biopsy will likely not be defined by competition between ctDNA and ncRNAs. Instead, the most informative assays will combine complementary molecular layers. ctDNA can identify genomic alterations and clonal evolution [13,14,15,16,17,18,19,20,21,22,23,24,25]; ncRNAs can reveal regulatory activity, tissue injury, immune modulation, and therapy-induced phenotypic change [26,27,28,29,30,31,32,33,34,35,36,37,38,39,40,41,42,43,44,45,46,47,48,49,50,51,52,59,60,61,62,63,64,65,66,67]; proteins and metabolites can report downstream functional consequences; EV markers can provide carrier and cellular-context information; and imaging or clinical metadata can anchor molecular signals to patient phenotype [20,21,22,23,24,25,166]. Recent literature increasingly emphasizes that this integration will rely on longitudinal, multimodal, and multi-omics datasets, supported by AI-enabled feature selection and clinically validated analytical workflows rather than by simply adding more analytes to existing panels [166,167,168].

In this integrated model, ncRNAs represent the functional transcriptomic layer of precision liquid biopsy [31,32,33,34,35,36,37,38,39,40,41,42,43,44,45]. Their greatest value may emerge when they are not treated as isolated biomarkers, but as context-dependent signals interpreted according to carrier, biofluid, biological process, and clinical question [59,60,123,124,125,126,127,166]. The field should therefore move from descriptive catalogues of differentially expressed RNAs toward clinically locked, mechanistically interpretable, and technologically robust signatures that are benchmarked across platforms, populations, and intended-use settings [73,74,75,76,77,78,79,154,158,166,167,168].

## 12. Conclusions

ncRNAs may represent the missing functional layer of cancer liquid biopsy. While ctDNA captures the genomic scars of cancer, ncRNAs capture ongoing biological activity. Their detection in blood and other body fluids offers opportunities for early detection, tumor classification, prognosis, treatment monitoring, MRD assessment, and prediction of therapeutic resistance. However, clinical value will depend on overcoming pre-analytical variability, analytical bias, biological confounding, and insufficient prospective validation.

The next generation of ncRNA liquid biopsy should account for carrier state, biological context, and molecular function. It should integrate RNA signals with genomic, proteomic, vesicular, cellular, imaging, and clinical information. Most importantly, it should move from exploratory biomarker discovery to standardized, multiplexed, prospectively validated assays that operate within real diagnostic pathways. ncRNA liquid biopsy should therefore be viewed not as an alternative to ctDNA, but as the functional layer that may complete precision oncology diagnostics.

## Figures and Tables

**Table 1:** Non-coding RNA (ncRNA) classes and their liquid biopsy relevance. Abbreviations: AI, artificial intelligence; circRNA, circular RNA; lncRNA, long non-coding RNA; miRNA, microRNA; piRNA, PIWI-interacting RNA; snoRNA, small nucleolar RNA; tRF, tRNA-derived fragment.

RNA Class	Liquid Biopsy Relevance
microRNAs	Stable and abundant disease-associated small RNAs; useful for early detection, tissue injury, prognosis, and therapy monitoring. Key challenges include normalization, hemolysis, extraction bias, and cohort reproducibility.
lncRNAs	Often tissue- or cancer-enriched regulatory RNAs; useful for tumor classification, prognosis, and resistance biology. Key challenges include low abundance, degradation, and assay sensitivity.
circRNAs	Structurally stable circular RNAs; promising robust biomarkers in plasma, urine, saliva, and stool. Key challenges include annotation, back-splice quantification, and platform comparability.
piRNAs/tRFs/snoRNAs/Y RNAs	Emerging high-dimensional small-RNA signatures; useful as cancer fingerprints and AI-assisted classifiers. Key challenges include biological interpretation and standardization.
Poorly annotated/orphan ncRNAs	Potentially cancer-informative non-canonical signals; useful for AI-driven early detection and subtype discovery. Key challenges include functional validation and clinical interpretability.

**Table 2:** Intended use scenarios for cancer ncRNA liquid biopsy. Abbreviations: AI, artificial intelligence; circRNA, circular RNA; ctDNA, circulating tumor DNA; EMT, epithelial–mesenchymal transition; lncRNA, long non-coding RNA; MRD, minimal residual disease; ncRNA, non-coding RNA.

Intended Use	Clinical Question and Validation Focus	Key Validation Challenges
Early detection	Question: Is cancer likely to be present before symptoms or imaging findings? Signal: broad high sensitivity ncRNA signatures, possibly multi-analyte. Endpoint: sensitivity and specificity in prospective screening or enriched-risk cohorts.	Low pre-test probability inflates false-positive burden; requires very high specificity and large prospective screening cohorts.
Tumor classification	Question: What is the likely tissue of origin or molecular subtype? Signal: tissue-enriched miRNA, lncRNA, circRNA, and AI signatures. Endpoint: correct tumor-source prediction and subtype discrimination.	Requires diverse benign/inflammatory comparators to avoid inflated apparent accuracy from case–control designs.
Prognosis	Question: Is the disease biologically aggressive? Signal: ncRNAs linked to EMT, angiogenesis, invasion, inflammation, or metastasis. Endpoint: progression-free survival, overall survival, or relapse risk.	Must add value beyond established clinicopathological variables; needs long follow-up and adjustment for confounders.
Treatment selection	Question: Which therapy is more likely to work? Signal: EV-associated or pathway-linked ncRNA signatures. Endpoint: response rate, durable benefit, immune response, or resistance emergence.	Requires demonstration that the signal predicts differential therapeutic benefit, not merely disease burden.
Monitoring/MRD	Question: Is residual or recurrent disease biologically active? Signal: longitudinal ncRNA changes combined with ctDNA and imaging. Endpoint: lead time to recurrence, relapse-free survival, and treatment adaptation.	Needs defined lead time over standard-of-care imaging/ctDNA and reproducible longitudinal assay performance.

**Table 3:** Comparative advantages and limitations of analytical technologies for ncRNA liquid biopsy translation. Abbreviations: Cas, CRISPR-associated; CRISPR, clustered regularly interspaced short palindromic repeats; DCL, Dynamic Chemical Labelling; DETECTR, DNA Endonuclease-Targeted CRISPR Trans Reporter; dPCR, digital polymerase chain reaction; PCR, polymerase chain reaction; RT-qPCR, reverse-transcription quantitative polymerase chain reaction; SERS, surface-enhanced Raman spectroscopy; SHERLOCK, Specific High-Sensitivity Enzymatic Reporter UnLOCKing.

Technology	Advantages	Limitations
RNA-seq/small-RNA-seq	Unbiased discovery; detects canonical and non-canonical/novel RNA species; broad multiplexing.	High cost per sample; complex bioinformatics; batch effects; longer turnaround time.
Targeted sequencing	Improved depth and reduced cost versus whole-transcriptome sequencing; flexible panel design.	Requires careful library preparation and normalization; panel content must be pre-specified, limiting discovery.
RT-qPCR	Sensitive, widely available, low cost per target, fast turnaround, familiar to clinical laboratories.	Low multiplexing; affected by reverse-transcription efficiency, primer specificity, and normalization uncertainty.
dPCR	Absolute quantification without a standard curve; high sensitivity and precision for selected targets.	Limited multiplexing capacity compared with sequencing or bead-based platforms; higher cost per target than qPCR.
Direct/PCR-free approaches (e.g., DCL)	Reduces extraction, reverse-transcription, and amplification-related bias; potential for simplified workflows.	Early-stage technology; oncology validation still limited; sensitivity for very-low-abundance targets not yet fully established.
CRISPR-Cas diagnostics (SHERLOCK/DETECTR)	Programmable target recognition; potential for portable, point-of-care formats.	Many implementations still require pre-amplification; quantitative performance in complex biofluids needs further validation.
SERS/plasmonic biosensors	Low sample consumption; potential for optical multiplexing; compatible with multiple analyte types (nucleic acids, proteins, EVs).	Substrate reproducibility, matrix interference, and cross-instrument calibration remain unresolved.
Nanopore/direct RNA sequencing	Long-read and potentially isoform- and epitranscriptomic-level information without amplification.	Higher input requirements, limited short-RNA capture, higher error rates in current clinical biofluid applications.

**Table 4:** Pre-analytical and analytical variables affecting ncRNA liquid biopsy. Abbreviations: dPCR, digital polymerase chain reaction; ncRNA, non-coding RNA; RT-qPCR, reverse-transcription quantitative polymerase chain reaction.

Variable Category	Impact and Mitigation
Sample collection	Tube type, anticoagulant, and time to processing can affect RNA degradation, blood-cell contamination, and carrier distribution. Mitigation: standardized collection and processing procedures.
Hemolysis and platelets	Red blood cell lysis and platelet contamination can falsely elevate blood-cell-derived miRNAs and other RNAs. Mitigation: hemolysis assessment and platelet-depletion protocols.
RNA isolation	Extraction kit, carrier enrichment, and input volume can bias recovery across RNA classes. Mitigation: spike-ins, process controls, and platform comparison.
Quantification	RT-qPCR, dPCR, sequencing, and hybridization assays have platform-specific sensitivity and bias. Mitigation: analytical validation and cross-platform benchmarking.
Data analysis	Normalization, batch effects, and model training can cause false discovery or poor generalization. Mitigation: locked pipelines, external validation, and transparent reporting.

## Data Availability

No new data were created or analyzed in this study. Data sharing is not applicable to this article.

## Abbreviations

The following abbreviations are used in this manuscript:

**Abbreviation**	**Definition**
AI	artificial intelligence
cfDNA	cell-free DNA
circRNA	circular RNA
ctDNA	circulating tumor DNA
DCL	Dynamic Chemical Labelling
dPCR	digital polymerase chain reaction
EMT	epithelial–mesenchymal transition
EV	extracellular vesicle
lncRNA	long non-coding RNA
miRNA	microRNA
MRD	minimal residual disease
ncRNA	non-coding RNA
PCR	polymerase chain reaction
piRNA	PIWI-interacting RNA
RT-qPCR	reverse-transcription quantitative polymerase chain reaction
snRNA	small nuclear RNA
snoRNA	small nucleolar RNA
tRF	tRNA-derived fragment
Cas	CRISPR-associated
CRISPR	clustered regularly interspaced short palindromic repeats
DETECTR	DNA Endonuclease-Targeted CRISPR Trans Reporter
SERS	surface-enhanced Raman spectroscopy
SHERLOCK	Specific High-Sensitivity Enzymatic Reporter UnLOCKing
